# Borrelial Lymphocytoma of the Ankle: An Unusual Presentation of Lyme Borreliosis

**DOI:** 10.7759/cureus.112334

**Published:** 2026-07-09

**Authors:** Bouchra Idrissi-Rhenimi, Syrine Hamada, Mariame Meziane, Nadia Ismaili, Benzekri Laila

**Affiliations:** 1 Dermatology, Ibn Sina University Hospital Center/Mohammed V University, Rabat, MAR

**Keywords:** ankle lesion, borrelial lymphocytoma, doxycycline, lyme borreliosis, pseudolymphoma, tick bite

## Abstract

Borrelial lymphocytoma is an uncommon cutaneous manifestation of Lyme borreliosis and is considered a localized reactive B-cell pseudolymphoma induced by infection with members of the *Borrelia burgdorferi* sensu lato complex. The condition most commonly affects the ear lobe, nipple-areolar region, and scrotum, whereas involvement of the ankle is rarely reported.

We report the case of a 47-year-old man who presented with a persistent erythematous papule adjacent to the lateral malleolus following a tick bite sustained approximately one year earlier. The lesion persisted despite several topical treatments. The patient did not recall a preceding episode of erythema migrans and had no extracutaneous manifestations suggestive of Lyme borreliosis. *Borrelia* serology performed by enzyme-linked immunosorbent assay (ELISA) revealed positive *Borrelia* IgG antibodies, which were subsequently confirmed by immunoblot. Histopathological examination of the skin biopsy demonstrated a dense dermal lymphoid infiltrate with reactive lymphoid follicles and numerous plasma cells, findings consistent with borrelial lymphocytoma in the appropriate clinicopathological context.

Treatment with doxycycline (100 mg twice daily for three weeks) resulted in marked clinical improvement and near-complete resolution of the lesion within a few weeks. No recurrence was observed during six months of follow-up.

This case highlights the rare occurrence of borrelial lymphocytoma involving the lateral malleolus, an unusual anatomical location that may delay diagnosis because of its atypical presentation. It also emphasizes the importance of clinicopathological correlation, integrating the clinical history, serological findings, and histopathological features, in establishing an accurate diagnosis and distinguishing borrelial lymphocytoma from other cutaneous pseudolymphomatous and lymphoproliferative disorders.

## Introduction

Lyme borreliosis is the most common tick-borne disease in Europe and is caused by spirochetes belonging to the *Borrelia burgdorferi* sensu lato complex [1,2]. Its clinical manifestations are heterogeneous and include early localized, early disseminated, and late-stage disease. The main cutaneous manifestations are erythema migrans, borrelial lymphocytoma (BL), and acrodermatitis chronica atrophicans [1,2].

BL is an uncommon cutaneous manifestation of Lyme borreliosis and is considered a localized reactive B-cell pseudolymphoma induced by *Borrelia* infection [1,3,4]. Clinically, it usually presents as a bluish-red papule, plaque, or nodule. The most frequently affected sites are the ear lobe in children and the nipple-areolar region or scrotum in adults [3-5].

Because of its rarity and variable clinical presentation, BL may be difficult to recognize, particularly when lesions occur at unusual anatomical locations. Histopathological examination, together with clinical history and serological findings, plays a crucial role in establishing the diagnosis and distinguishing BL from cutaneous lymphomas and other pseudolymphomatous disorders [3,6]. Reports of lesions involving the ankle remain exceptionally rare, and awareness of such atypical presentations may facilitate earlier diagnosis and appropriate treatment. We report a histologically confirmed case of BL adjacent to the lateral malleolus, with complete clinicopathological correlation and marked clinical improvement following doxycycline therapy.

## Case presentation

A 47-year-old man was referred to our dermatology department for evaluation of a persistent lesion located adjacent to the lateral malleolus of the left ankle. The patient reported a tick bite sustained during forest exposure approximately one year before presentation. Several months after the bite, he gradually developed a mildly pruritic papular lesion at the same site. The lesion persisted despite several topical treatments prescribed elsewhere, without significant clinical improvement.

The patient did not recall a preceding episode of erythema migrans and denied constitutional symptoms, arthralgia, cardiac manifestations, or neurological complaints. *Borrelia* serology performed approximately one year after the reported tick bite using enzyme-linked immunosorbent assay (ELISA) revealed positive *Borrelia* IgG antibodies, which were subsequently confirmed by immunoblot, consistent with previous exposure to *Borrelia* species.

Clinical examination revealed a solitary erythematous papule with central umbilication located adjacent to the lateral malleolus. The lesion measured only a few millimeters in diameter and was soft on palpation (Figure 1). Dermoscopic examination demonstrated a central crusted umbilication surrounded by a homogeneous pink structureless area (Figure 2).

**Figure 1:**
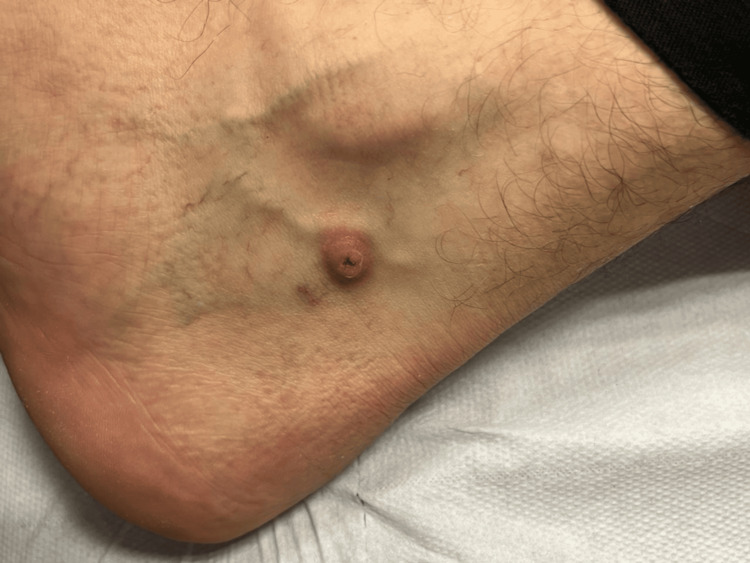
Clinical appearance of borrelial lymphocytoma at presentation Solitary erythematous papule with central umbilication located adjacent to the lateral malleolus of the left ankle before treatment.

**Figure 2:**
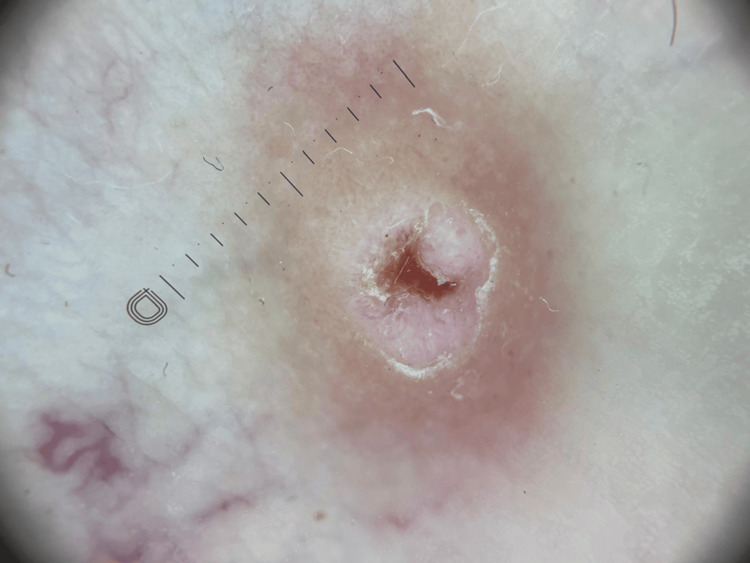
Dermoscopic features of the lesion Dermoscopic examination showing a central crusted umbilication surrounded by a homogeneous pink structureless area.

Given the history of tick exposure and the chronic course of the lesion, a skin biopsy was performed. Histopathological examination demonstrated a dense, diffuse lymphocytic infiltrate involving the superficial and deep dermis, with numerous reactive lymphoid follicles containing prominent germinal centers. The infiltrate consisted predominantly of small mature lymphocytes admixed with plasma cells and histiocytes, without cytological atypia, findings consistent with BL in the appropriate clinicopathological context.

The patient was treated with doxycycline 100 mg twice daily for three weeks. Follow-up examination demonstrated marked clinical improvement. Within a few weeks after completion of treatment, the lesion had almost completely resolved, leaving only minimal residual erythema (Figure 3). No recurrence was observed during the six-month follow-up.

**Figure 3:**
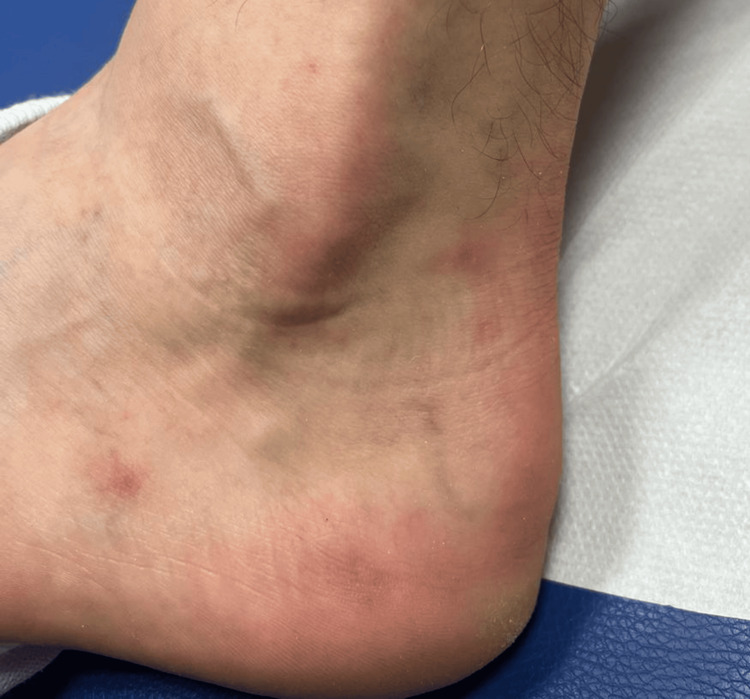
Clinical outcome after doxycycline therapy Resolution of the lesion after three weeks of doxycycline treatment, leaving minimal residual erythema.

## Discussion

BL is an uncommon manifestation of Lyme borreliosis and represents a localized reactive B-cell pseudolymphoma associated with infection by members of the *B. burgdorferi* sensu lato complex [1,2]. In Europe, *Borrelia afzelii* is considered the predominant causative species [7].

The main interest of our case lies in its unusual anatomical location. In the largest adult series published to date, Maraspin et al. analyzed 144 adult patients with BL and reported that lesions were located on the breast in 73.6% of cases, on the ear lobe in 18.8%, and at other anatomical sites in only 7.6% [4]. Likewise, Strle et al. described 36 patients, with the vast majority of lesions involving the ear lobe or mammary region, whereas only four cases occurred at uncommon locations [5]. These findings emphasize the rarity of ankle involvement and explain why lesions arising near the lateral malleolus may not immediately suggest Lyme borreliosis.

The clinical appearance of our patient’s lesion was also atypical. BL is classically described as a bluish-red infiltrated plaque or soft nodule measuring up to several centimeters in diameter. In contrast, our patient presented with a small erythematous papule measuring only a few millimeters. Such an appearance may mimic arthropod bite reactions, adnexal tumors, molluscum contagiosum, keratoacanthoma-like lesions, or other inflammatory dermatoses, potentially delaying diagnosis [3].

Another noteworthy feature was the absence of erythema migrans. Maraspin et al. reported concomitant erythema migrans in 72.2% of patients with BL, whereas extracutaneous manifestations of Lyme borreliosis were documented in only a minority of cases [4]. Our patient had neither erythema migrans nor systemic manifestations, making the diagnosis less clinically obvious and highlighting the importance of clinicopathological correlation.

Serological findings further supported the diagnosis. Positive *Borrelia* IgG antibodies detected approximately one year after tick exposure were consistent with previous infection. Persistent IgG seropositivity has been well documented and may remain detectable for several years after exposure; therefore, serological results should always be interpreted in conjunction with clinical and histopathological findings [1,2].

Histopathological examination played a pivotal role in establishing the diagnosis. In our case, the biopsy demonstrated a dense dermal lymphocytic infiltrate with reactive germinal centers, numerous plasma cells, and eosinophils, findings that are characteristic of BL and helped distinguish it from cutaneous lymphoma and other pseudolymphomatous disorders [3,6].

The favorable response to doxycycline further reinforced the diagnosis. Previous studies have shown excellent clinical outcomes following standard antibiotic regimens recommended for Lyme borreliosis [4,5]. In our patient, doxycycline administered at 100 mg twice daily for three weeks resulted in near-complete regression of the lesion, with no evidence of recurrence during six months of follow-up.

Overall, the uniqueness of this case lies in the unusual lateral malleolar localization, the absence of preceding erythema migrans, and the atypical papular clinical presentation, while the positive *Borrelia* IgG serology, characteristic histopathological findings, and favorable response to doxycycline supported the diagnosis. Recognition of such atypical presentations may facilitate earlier diagnosis and appropriate treatment of this rare manifestation of Lyme borreliosis.

## Conclusions

BL should be considered in the differential diagnosis of persistent papular lesions occurring at atypical anatomical locations in patients with a history of tick exposure. Histopathological examination remains essential for establishing the diagnosis by demonstrating a dense dermal lymphocytic infiltrate with reactive germinal centers, numerous plasma cells, and eosinophils, while also distinguishing BL from other pseudolymphomatous disorders and primary cutaneous lymphomas.

This case highlights a rare presentation of BL involving the lateral malleolus, without preceding erythema migrans or extracutaneous manifestations of Lyme borreliosis. Recognition of such atypical presentations may facilitate earlier diagnosis and appropriate antibiotic therapy, thereby reducing diagnostic delay and improving patient outcomes.
